# Supplementary material for: The governmental ranking of class and the academic performance of Indian adolescents
Source: PLoS One. 2020 Nov 2;15(11):e0241483. doi: 10.1371/journal.pone.0241483 (PMC7605817; doi:10.1371/journal.pone.0241483)
Supplement: Attachment — Submitted filename: Review report.pdf [file pone.0241483.s002.pdf]

Review Report for paper titled “Governmental Ranking of Class and Academic Performance of Indian Adolescents”

Anwesha Bandyopadhyay

January 21, 2020

This paper studies the impact of caste system on academic performance of Indian students in grades 10 and 11 who study in similar school environment. I found the topic very interesting and relevant. Academic performance in these grades are very important for Indian students and likely to affect the subjects they chose and the schools/colleges they choose for further study. The results are also very thought provoking and can have significant impact on policies directed towards backward communities in India.

Here are some more suggestions to help improve the quality of the paper.

Major points:

- Describe the difference between caste system and government class in a bit more detailed manner. The authors can either incorporate this description in the introduction or put it in a footnote. The authors can also incorporate history on caste system to explain why this system is so deep rooted in India even till this day. Of course, apart from history there are other factors such as education that has kept this system alive in India till this day. This will help the readers who are not well aware of this difference.
- There are several examples of caste-based discrimination in India. It includes, honour killing in case of inter-caste marriages, bullying students from lower castes in higher educational institutions, untouchability, refusal to eat food cooked by people belonging to lower castes etc. Using a wide array of examples from different spheres of life in India will help readers understand the extent of discrimination and the impact on the life of people belonging to lower castes due to this discrimination.
- Explain why choose Kerala and Madhya Pradesh. This is important and interesting since Kerala has more liberal outlook to caste, religion etc. compared to Madhya Pradesh which is very traditional.
- In the methodology section, explain in brief the nature of assessment that students were asked to do.
- In the section statistical analysis, please check line 5 (in that section). I believe the a little rewording and rearranging the words can make the sentence construction better.
- Since Kerala and Madhya Pradesh are culturally quite different, it will be good if the authors can control for state characteristics in their regressions. They can either use state fixed effects if possible or can use data on caste based crimes. Such data can be found in the website of National Crime Records Bureau(India). Also, the authors can run two separate regressions for the two states and compare the results.

- Were the students asked if they take private tuition and if yes, then for how many subjects? Indian students have a tendency to go for private tuition and that can impact their academic performances.

Minor Point:

- It would have been interesting if the authors could include data on students in the arts and humanities stream. In India, there is a bias against students in humanities.
