# Supplementary material for: The governmental ranking of class and the academic performance of Indian adolescents
Source: PLoS One. 2020 Nov 2;15(11):e0241483. doi: 10.1371/journal.pone.0241483 (PMC7605817; doi:10.1371/journal.pone.0241483)
Supplement: Attachment — Submitted filename: PLOS_ONE__resubmit_review (1).pdf [file pone.0241483.s004.pdf]

Review Report for resubmitted version of the paper titled "Governmental Ranking of Class and Academic Performance of Indian Adolescents"

Anwesha Bandyopadhyay

July 1, 2020

This paper studies the impact of caste system on academic performance of Indian students in grades 10 and 11 who study in similar school environment. The authors have shown immense sincerity in incorporating suggestions they obtained after the review process. In cases the authors chose not to incorporate the suggestions, they have provided valid and acceptable reasons for their choices. I believe this study will be an important addition to the existing literature in Development Economics and Economics of Education. Please accept my best wishes.

I just have one suggestion.

In page 10, in the line "A multilevel model was then set up to assess the relationship between caste affiliation school performance for all six schools combined, once again controlling for the above mentioned five covariates.", there is a small error. I believe the correct way to write this sentence will be:
A multilevel model was then set up to assess the relationship between caste affiliation 'AND' school performance for all six schools combined, once again controlling for the above mentioned five covariates.
