# Supplementary material for: The governmental ranking of class and the academic performance of Indian adolescents
Source: PLoS One. 2020 Nov 2;15(11):e0241483. doi: 10.1371/journal.pone.0241483 (PMC7605817; doi:10.1371/journal.pone.0241483)
Supplement: Attachment — Submitted filename: 3-Response to reviewers 2.docx [file pone.0241483.s003.docx]

**Response to Reviewer #2:**
The manuscript deals with the relationship between the government classes and academic performance (AP) of the students of class X and XI in two states of India. The topic is of immense importance and quite under-researched. While the paper has potential, it has not fully utilized the data that might have been available to the authors. The literature survey needs to be broadened and application of literature to the findings and discussion needs tightening. Following are my specific comments

**Response to the Comment**

Thank you very much for your highly helpful comments. We have taken utmost care to incorporate all your suggestions and to respond to all of observations. We have revisied our manuscript accordingly. We have modified the introduction, method, results and discussion on the basis of your comments. If you deem our responses as not fully fitting or in case, we missed your point, we would of course be willing to address them further.

**Comment 1:** Introduction
What are the enrolment proportions and drop-outs in secondary and senior secondary school for the government classes and their performance at the national level? See MHRD report (ESAG).

**Authors’ response to reviewers’ comment**

Thank you very much for the observation and for providing a relevant reference. This information is definitely useful while discussing the difference in academic performance across the governmental classes. However, we do not have data about the proportions of enrollment, drop-outs and academic performance of all the three governmental classes in the Ministry of Human Resource Developmentestimates (ESAG 2018). The report mentions theoverall data and then separate data for only SC-ST categories. There are no separate data on the Other Backward Castes (OBCs) and General Class. Based on the ESAG 2018, at Senior Secondary school, out of the 24.7 students who get enrolled, 5.9 are from the SC-ST category, which is slightly less than their nationalproportion of the SC-ST category in India (27.5%). Similarly, out of 19.4 million students who enrolled for secondary school board examinations in 2016, 4.9 million were from SC-ST. As per the 68^th^ Round of the National Sample Survey (NSS 2011-12), the rate of educational attendance in India in the age range of 5-14years were 88-89% for SC-ST, 90% for OBC, and 93% for GC. For the age range of 15-19 years, the rate of attendance dropped and the gap between the three categories widened to 54-57% for SC-ST, 64% for OBC, and 71% for GC. Drop out rates are higher for students from the SC-ST categories (19.4% for SC and 24.7% for ST) as compared with drop out rates of all students (17.1%).More importantly, as per the MHRD report the proportion ofstudents who passed the secondary school board examinations in 2016 was lower for the SC-ST categories of students (73% for SC and 65% for ST) when compared to all students (78.7%). Though this document does not mention GC and OBC, nonetheless, this information could be indicative of a trend that students from lower classes perform poorly at secondary schools across students of Central and State boards. Similar trend is observed also in the NSS 2011-12. In separate estimates for the States, in July 2012 arelatively lower rate of persons from the lower classes completed secondary education in Madhya Pradesh (SC-ST = 7%, OBC = 10%, GC = 15%), as well as Kerala (SC-ST = 15%, OBC = 18%, GC = 23%).

Revision on page 4

***According to the National Sample Survey (NSS 2011-12, 68^th^ Round), the proportion of persons SC-ST in India is 27.5%, OBC 44% and GC 28.5% (15). Based on the yearly report of Ministry of Human Resource Development (ESAG 2018), at Senior Secondary school, out of the 24.7 students who gott enrolled, 5.9 were from the SC-ST category, which is slightly less than their national the proportion (27.5%) in India (16). Similarly, out of 19.4 million students who enrolled for secondary school board examinations in 2016, 4.9 million were from SC-ST. As per NSS 2011-12, the rate of educational attendance in India in the age range of 5-14years were 88-89% for SC-ST, 90% for OBC, and 93% for GC. For the age range of 15-19 years, the rate of attendance dropped and the gap between the three categories widened to 54-57% for SC-ST, 64% for OBC, and 71% for GC (15). Drop out rates are higher for students from the SC-ST categories (19.4% for SC and 24.7% for ST) as compared with drop out rates for all students (17.1%) in India (16).***

Revision on page 5

***According to the ESAG 2018 report (16), the proportion of students who passed the secondary school board examinations in 2016 was lower for the SC-ST categories of students (73% for SC and 65% for ST) when compared to all students (78.7%). Though this document does not mention GC and OBC, nonetheless, this information could be indicative of a trend that students from lower classes perform poorly at secondary schools across students of Central and State boards. Similar trend is observed also in the NSS 2011-12 (15). In separate estimates for the States, in July 2012 a relatively lower rate of persons from the lower classes completed secondary education in Madhya Pradesh (SC-ST = 7%, OBC = 10%, GC = 15%), as well as Kerala (SC-ST = 15%, OBC = 18%, GC = 23%) (15).***

References added:

**National Sample Survey, 68^th^ Round (July 2011 – June 2012). Employment and Unemployment Situation among Social Groups in India. Ministry of Statistics and ProgrammeImplementation, Government of India. 2015.** [**http://mospi.nic.in/sites/default/files/national_data_bank/pdf/NSS_68Round-563.pdf**](http://mospi.nic.in/sites/default/files/national_data_bank/pdf/NSS_68Round-563.pdf)

**Educational Statistics at A Glance. Ministry of Human Resource Development, Government of India. ESAG 2018.** [**https://mhrd.gov.in/sites/upload_files/mhrd/files/statistics-new/ESAG-2018.pdf**](https://mhrd.gov.in/sites/upload_files/mhrd/files/statistics-new/ESAG-2018.pdf)

**Comment 2:** Literature survey
Bourdieu’s theory of ‘cultural capital’ is useful here to explain differences in academic achievement based on socio-economic status.

**Authors’ response to reviewers’ comment**

Thank you very much for your valuable suggestion. We have revised the discussion in the manuscript to incorporate mention of how social and cultural capital is transferable to economic capital and hence how the caste system can disadvantage the students from lower castes economically.

Revision on page 5

***Bourdieu (22) proposed an elaborate theory of social, economical, and cultural capitals. According to him possessing economic resources (economic capital) contributes to increased social connection and influence (social capital). In turn, social capital may facilitate increased educational opportunities/achievement, which is part of the cultural capital (22, 23). In the Indian context, caste affiliation along with the financial condition of the family constitutes a major part of students’ socioeconomic and cultural capital (24, 25). Caste affiliation may determine to some extent the socioeconomic status of the family (11). Also, economic status irrespective of caste/class affiliation may determine academic achievement (17, 19). Gupta (26) found evidence for a differential influence of social status and economic status on academic performance in Indian college students. While students from lower castes were more likely to perform poorly in academics, students from lower economic status were not more likely to perform poorly.***

References added:

**Bourdieu, P. The forms of capital. In J. Richardson (Ed.) Handbook of Theory and Research for the Sociology of Education (New York, Greenwood). 1986: 241-258.**

**Huang, X. Understanding Bourdieu - Cultural Capital and Habitus. Rev. Eur. Studies. 2019: 11(3), 45-49. doi: 10.5539/res.v11n3p45**

**Comment 3:**

‘Alternately, the associated socio-economic status may determine academic achievement, irrespective of caste/class affiliation.’ Please give an example/reference.

**Authors’ response to reviewers’ comment**

This statement means that having higher/lower financial and social status may lead to access to better/poorer schooling and additional coaching opportunities, and lead to better/poorer academic achievement. As you have already suggested, the concept of different forms of capitals (Bourdieu, 1986) and their relations can explain this. The manuscript has been modified by providing references for this statement.

**Comment 4:**

The authors might benefit from the following article that deals with the relationship between caste and academic performance in higher education in India:
Namrata Gupta (2019) Intersectionality of gender and caste in academic performance: quantitative study of an elite Indian engineering institute, Gender, Technology and Development, 23:2, 165-186.<https://www.tandfonline.com/doi/abs/10.1080/09718524.2019.1636568>

**Authors’ response to reviewers’ comment**

Thank you very much for suggesting this reference to improve the literature survey. The manuscript has been modified to incorporate reference to this article. The referred study found that while students from lower castes were more likely to perform poorly in academics, students from lower economic status were not more likely to perform poorly.

Revision on page 5

***Gupta (26) found evidence for a differential influence of social status and economic status on academic performance in Indian college students. While students from lower castes were more likely to perform poorly in academics, students from lower economic status were not more likely to perform poorly.***

**Comment 5:**

Some of the references are not specific and does not lead the reader to the source. For instance: ‘India Go. Ministry of Social Justice and Empowerment, Government of India 2018, Sep’. Which document are you referring to?

**Authors’ response to reviewers’ comment**

Thank you very much for bringing to notice this error in referencing. In the revised manuscript, we have also gone through all references and ensured specificity as you have suggested.

The mentioned reference is corrected as:

**Social Justice AR 2018-19. Ministry of Social Justice and Empowerment, Government of India 2018** [**http://socialjustice.nic.in/writereaddata/UploadFile/Social_Justice_AR_2018-19_English.pdf**](http://socialjustice.nic.in/writereaddata/UploadFile/Social_Justice_AR_2018-19_English.pdf)

**Comment 6:**

On Pg. 10: ‘Though Indian studies have examined self-esteem and life satisfaction in the context of educational outcomes, …’ What Indian studies are you referring to?

**Authors’ response to reviewers’ comment**

Thank you very much for the comment. In the revised manuscript we haveprovided the references for this part of the statement. The references are (15) and (29).

**Comment 7: Methods**

We need more information about schools. Are they government, private or mixed schools? What is their language of instruction?
Were there any difficulties for the participants in filling up the questionnaire and how were they handled? For instance, was the assessment administered in English? If yes, could the students understand the language particularly in the self-esteem and life satisfaction scales?
What is a z-score?

**Authors’ response to reviewers’ comment**

Thank you very much for this comment. We have revised the methods section and the suggested details regarding participants and procedure have been incorporated. Since we conducted the study only in urban/semi-urban English medium schools, the questionnaires were administered in English. Students were assisted by their respective Eglish teachers in responding to the questionnaires.

The raw scores of academic performances from different streams of schools were converted to z-scores for making them comparable.

Revised on page 7

***The participating schools from the State of Madhya Pradesh followed CBSE syllabus (Central Board of Secondary Education) and Kerala schools followed State Board of Education (SBE) syllabus. The XI grade students were recruited from two different streams, i.e. science and commerce. These schools were from the private sector and English was the medium of instruction in all these schools*.**

Revised on page 9

***The assessments were administered during school hours and in classrooms. Students were given 30 minutes to complete the assessment. The medium of assessment was English since all the participants were from English medium schools.***

**Comment 8a: Results**

P. 14: ‘Predicted school performance values were 45.5 (±3.6), 49.1 (±3.3), and 51.4 (±3.3) for low, medium and high-class levels respectively, and were thus increasing with increasing levels of governmental class.’ Please specify if these are mean values taking all the schools together. Also, in the column on AP, there does not seem to be a linear relationship between class categories and AP, since, SC/ST average is 44.5, OBC 43.0 and GC 53.8. Please explain.

**Author’s response to reviewers’ comment**

We distinguish between AP mean values based on descriptive statistics (mean values across all students and schools by caste-class) and predicted mean values based on a multilevel model with caste taken as a factor with three levels (low, medium, and high). In both cases, all schools were included to compute means.

In addition (and independent of the computation of means as explained above), we set up an apriori hypothesis in which we wanted to test whether AP linearly increased with higher caste class (from SC-ST to OBC to GC). To this end we used caste-class as a linear predictor in a separate multilevel model.

**Comment 8b**

In Table 2, AP, SES and SWLS numbers for various categories are out of what? AP seems to be percentage, but the rest?

**Authors’ response to reviewers’ comment**

Thank you for making these observations. These numbers are Mean (SD) as given on the Table description on top [**Table2:** Descriptives: Academic performance, self-esteem and life satisfaction Mean (SD)]

**Comment 9: Discussion**

P. 16: Line: ‘Also, an increase of family income may not necessarily correspond to better educational outcomes for the students of low social class. In other words, the educational disadvantage that we observe in our results in the lower governmental class, may not be equated with economic disadvantage.’ Please state the reason for this.

**Authors’ response to reviewers’ comment**

Thank you very much for making these careful observations. That social backwardness may affect educational outcome independent of financial status is an argument that is presented in the introduction with supportive references(page5). Further, the results also point in this direction because caste affiliation contributed to academic performance when family income was controlled for. Additionally, to support this argument further, here we have added a descriptive observation that in our sample a relatively higher proportion of OBC class reported higher family income when compared with GC (This is only an additional observation and not a statistical evidence). In the revised manuscript these two sentences (‘Also, an increase of family income.... with economic disadvantage.’ have been since the argument is discussed elsewhere.

**Comment 10**

P. 16: ‘In a study (40) conducted in the state of Haryana in which students from a socioeconomically advanced OBC subgroup were included, the academic performance of these OBC students was found to be on par with the General Class.’
This statement appears flawed as the Yadav and Chahal, the authors of the paper cited here state in their own paper: ‘The reserved categories include OBC’s which is socially backward but economically prosperous category.’ (p.36: Yadav and Chahal 2016). Hence, Yadav and Chahal seem to have studied OBCs that are socially backward and cannot be referred to as ‘socioeconomically advanced’.

**Authors’ response to reviewers’ comment**

Thank you very much for carefullyidentifying this error. The statement has been corrected as “economically backward”. The phrase “socially backward families which are economically advanced” in the next sentence would further clarify it.

**Comment 11**

P. 14: Line: ‘In one school (KL2), academic performance was highest in the highest class (GC) and lowest in the lowest class (SC-ST) (p<.001) while in another school (KL3), academic performance also varied among classes, albeit being lowest for the medium class (OBC) (p=0.014). For the remaining four schools, no significant differences in academic performance among the three class levels were found…’
As your Table 1 shows, schools KL1 and MP2 have either a single or no SC/ST students. Hence the claim that: ‘For the remaining four schools, no significant differences in academic performance among the three class levels were found…’ does not seem pertinent. Instead it will be better to show the relationship between caste and AP for GC and OBC for all the schools. The status of the OBCs in Kerala and MP should also be discussed and related to the findings.

**Authors’ response to reviewers’ comment**

Thank you very much for this observation. The main focus of the study is to look at the overall sample (N=858) in the three categories. Examining of separate schools is only additional and not part of the design or objectives of the study. Since we planned to include the whole classroom while sample recruitment, the actual presence of the three categories of classes in these schools are reflected in the sample. Thus, KL1 and MP2 have either a single or no SC-ST students, and MP2has only 6 and MP3 has only 7 OBC students. GC are relatively better represented in all six schools. It may be interesting to notice that in KL2 where all three categories have sizeable representation, academic performance was highest in the highest class (GC) and lowest in the lowest class (SC-ST) (p<0.001), which is similar to our results for the overall data. As you have suggested, we have modified the text by omitting the statement ‘For the remaining schools...’ andour focusinstead will beon the three categories for all schools. Besides, as suggested, we also elaborate on the status of the govt classesin Kerala and MP.

Revised section on page 8

***According to NSS 2011-12, 93% population in Kerala as compared to 72% in Madhya Pradesh are literate. The two States also differ regarding the proportion of the three govt. classes. Kerala has only 9.5% persons of SC-ST, 65% OBC, and 25% GC, compared to 39% SC-ST, 42% OBC, and 19% GC in Madhya Pradesh. In Madhya Pradesh, there is a lower rate of persons who complete secondary education as well as lower gap across the three categories (SC-ST = 7%, OBC = 10%, GC = 15%), as compared with Kerala (SC-ST = 15%, OBC = 18%, GC = 23%). Thus, our sample had a lower representation of the lowest class (SC-ST) because their proportion is less than 10% in Kerala and only 7% of the SC-ST studied till Secondary School in Madhya Pradesh.***

Revised section on page 11

***Only in one school (KL2), there was a sizeable representation (See Table 1) of all the three govt. classes. For the other 5 schools, there was no proportionate/adequate representation of all three categories in each school when taken separately. Thus, when school KL2 was separately examined, it was found that academic performance was highest in the highest class (GC) and lowest in the lowest class (SC-ST) (p<.001). Assuming a linear functionality between governmental classes and academic performance, we found a positive association in this school (KL2: β=4.12, SE=1.23, t=4.17, p<0.001)where the students showed increasing academic performance with increasing class levels. For the other five schools, a significant pattern of association could not be observed (Table 5).***

Revised sections on page 14-15

***For instance, according to NSS 2011-12 (15), one in four SC-ST households had no literate family member of age 15 years and above in rural area, as compared to one in ten households in urban area. On comparison, among OBC, 18% households in rural areas compared to 7 per cent in urban areas and among GC, 11% in rural areas compared to 3% in urban areas had no literate member of age 15 years and above (15). The persons from the higher class were more likely to live and study in urban area as compared to the lower classes. Also, family members in the higher class were much more likely to have self-employment or salaried jobs as compared to the members of the lower classes who work mostly in the primary sector (15). Thus, the role of (lack of) education, social exposure, career aspirations and achievement motivation in relation to the caste-based social identity must be examined in future studies.***

***According to NSS 2011-12 (15), in Kerala, there is a higher rate of persons who complete secondary education as well as lower gap across the three categories (SC-ST = 14-16%, OBC = 18%, GC = 23%), as compared with Madhaya Pradesh (SC-ST = 5-8%, OBC = 10%, GC = 15%). Thus, although Kerala is relatively higher on social indicators (15, 51), the results for KL2 (Table 5) was similar to the overall sample. Hence, the difference among the classes across the States in India as well as the influence of the specific context of the States need to be also examined for a better understanding of the association of caste affiliation and academic performance.***

**Comment 12**

Based on the literature, the authors might like to discuss as to why there is a relationship between government classes/caste and the AP.

**Authors’ response to reviewers’ comment**

Thank you very much for the suggestion and our discussion has been modified to incorporate it. In this study, we have not examined the factors that might have contributed to the relationship between the classes and AP. Hence, after corroborating our results by refering to other studies, we go on to make hypothetical speculations on possible reasons for the relationshipin three paragraphs.Following are someof the reasons that we have discussed in the original manuscript:

1) The reason for our finding must be related to social status over and above the potential influence of economic status (family income)as well as some of the psychological factors (self-esteem and general wellbeing/life satisfaction) that were controlled for. In other words, reasons specific to belonging to a household of backward caste must have contributed to the poorer academic performance of the students from the backward castes/classes. We leave it to future studies to examine what aspects of caste identity and social status influence academic performance. In our revised manuscript, we include that limited social exposure and minimal aspiration/achievement motivation are likely to influence academic performance.

2) The influence of social backwardness on education may be increased by associated economic backwardness. We refer to study which found that socially backward students who were economically advanced performed well in academics on par with GC students (The manuscript has been modified to bring emphasis on this argument).

3) Yet another reason could be English language proficiency since our participants were from schools with English as medium of instruction. Students from the lower classes are much more likely to come from families with parental lack of education and lack of proficiency in English language. This is likely to influence their academic performance.

The manuscript has been modified to strengthen these arguments.

Revised section on page 14-15

***The reason for our results must be linked to the influence of social status over and above the potential influence of economic status (family income) as well as some of the psychological factors (self-esteem and general wellbeing/life satisfaction) that were controlled for. Factors specific to belonging to a household of backward caste must have contributed to the poorer academic performance of the students from the backward castes/classes. Students from the backward castes are likely to be low on educational and career aspirations. This could be so because the household of the students may have less understanding of or exposure to educational and career opportunities different from their traditional caste-based social and occupational roles. Hence, the potential role of caste-based social identity and its relation to (lack of) social exposure, career aspirations and achievement motivation must be examined in future studies.***

***The factors that influence academic performance may be diverse and complex for all the numerous subgroups placed under the three governmental classes. Some of the social subgroups under OBC category have high economic prosperity and respectable social status in some states/districts of India. In a study (27) conducted in the state of Haryana in which students from an economically advanced OBC subgroup were included, the academic performance of these OBC students was found to be on par with the General Class. Thus, the influence of social backwardness on education may be increased by associated economic backwardness. Hence, the differential influence of governmental class for students from socially backward families which are economically advanced versus economically backward needs to be examined in the Indian context.***

***Another important factor that may have influenced the students’ academic performance reason could be English language proficiency since our participants were from schools with English as medium of instruction. Students from the lower classes are much more likely than their counterparts to come from families with parental lack of education and lack of proficiency in English language. This is likely to influence their academic performance.***

**Comment 13**

‘(11) observed that students from lower socioeconomic status are likely to have a positive influence...’
Do you mean to say ‘…. likely to be positively influenced…’? Please replace (11) by the last name of the referred author.

**Authors’ response to reviewers’ comment**

Thank you for making this suggestion and pointing out the error. Yes. We meant to say “... likely to be positively influenced”.Also, (11) is replaced with the author’s last name.

**Comment 14**

‘This would imply that the gap in academic performance between students from higher and lower governmental classes are likely to widen if students from the schools of the governmental sector and from the rural background are investigated.’
Because??

**Authors’ response to reviewers’ comment**

We collected our data from urban areas where all the three class categories of students are likely to study together in the same schools. This co-education is likely to improve the academic performance of the lower class as observed in the referred article. On the other hand, in rural areas and especially in schools of government sector which provide free education, students from higher class are seldom represented and hence there would not be possiblity for co-education of higher and lower classes. Hence, we specualte that without the potential advantage of co-education, these students are likely to perform poorer academically, if compared with students of lower class who study with students of higher class. The text has been modified to make this argument more lucid.

Revised text on page 16

***Thomson (18) observed that students from lower socioeconomic status are likely to be positively influenced when they study along with students of higher socioeconomic status and are likely to perform better academically. Thus, if studying together with students from higher governmental class is advantage for students from lower class, then it would imply that we may find a greater gap in academic performance if we include students from rural schools who are mostly from the lower classes and who are less likely to study with students of higher class.***
