# Supplementary material for: The governmental ranking of class and the academic performance of Indian adolescents
Source: PLoS One. 2020 Nov 2;15(11):e0241483. doi: 10.1371/journal.pone.0241483 (PMC7605817; doi:10.1371/journal.pone.0241483)
Supplement: Attachment — Submitted filename: Response to reviewers.docx [file pone.0241483.s005.docx]

**Reviewer #1:**

In page 10, in the line "A multilevel model was then set up to assess the relation-ship between caste affiliation school performance for all six schools combined, once again controlling for the above mentioned five covariates.", there is a small error. I believe the correct way to write this sentence will be: A multilevel model was then set up to assess the relationship between caste affiliation `AND' school performance for all six schools combined, once again controlling for the above mentioned five covariates.

***Response: We have corrected the sentence accordingly.***

**Reviewer #2:**

While there is a detailed discussion of results on academic performance and its relation to socio-economic status, there is no discussion on the two other measures, self-esteem (SES) and satisfaction with life (SWLS). How were these related to academic performance? Also, what were the gender differences in performance with respect to their caste?

***Response: We have included the calculations and results for SES, SWLS as well as gender, family income and age in the results section and also addressed these findings in the discussion section with two added sentences.***

The Discussion needs to engage with Bourdieu, that is, how far it affirms the theory of cultural/social capital.

***Response: While we fully agree that our manuscript is possibly relevant to the area of sociology, we must admit that none of the authors has any backgrpund or experience in this discipline. Thus, any informed discussion of Bourdieu would appear assumptive from our side. We hope that this is not a major problem, but of course we hope that our paper and the results will be picked up by relevant disciplines, such as sociology, educational sciences and policy studies.***

Line in Introduction: 'Based on the yearly report of Ministry of Human Resource Development (ESAG 2018), at Senior Secondary school, out of the 24.7 students who got enrolled, 5.9 were from the SC-ST category..." What are 24.7 and 5.9? percentages?

***Response: Yes, the % was missing and we have corrected the sentence accordingly.***

The line in the Discussion 'Thus, the influence of social backwardness on education may be alleviated by better economic condition and worsened by economic backwardness.' is confusing. It might either be modified or deleted.

***Response: We deleted the sentence.***

The last line in the Discussion section: 'then it would imply that we may find a greater gap in academic performance if we include students from rural schools who are mostly from the lower classes and who are less likely to study with students of higher class'. This line is also not clear. "include" where?

***Response: We deleted the sentence.***
